# Primary small cell carcinoma of the breast: a case report

**DOI:** 10.1186/s13256-017-1467-0

**Published:** 2017-10-19

**Authors:** Iman Abou Dalle, Jaber Abbas, Fouad Boulos, Ziad Salem, Hazem I. Assi

**Affiliations:** 1 Department of Internal Medicine, Hematology-Oncology Division, American University of Beirut Medical Center, Beirut, Lebanon; 2 Department of Surgery, American University of Beirut Medical Center, Beirut, Lebanon; 3 Department of Pathology and Laboratory Medicine, American University of Beirut Medical Center, Beirut, Lebanon

**Keywords:** Small cell carcinoma, Breast cancer, Neuroendocrine, Chemotherapy

## Abstract

**Background:**

Neuroendocrine breast cancer is a rare entity that was defined in 2003 by the World Health Organization as a separate breast cancer subtype. The diagnosis of neuroendocrine breast cancer requires the presence of neuroendocrine features in at least 50% of malignant cells, the exclusion of non-mammary primary tumors, as well as the presence of an *in situ* component in breast histology. The treatment and prognosis of neuroendocrine breast cancer are still not well established. Small cell carcinoma of the breast is a subtype of neuroendocrine cancer, resembling small cell carcinoma of the lung. It has a very poor prognosis and warrants treatment with platinum-based chemotherapy.

**Case presentation:**

We herein report the case of a 47-year-old white woman with a left breast mass that was found to be an early-stage, high-grade small cell carcinoma of the breast. Positron emission tomography-computed tomography imaging excluded any other primary disease. Our patient underwent a left total mastectomy with sentinel lymph node biopsy and received cisplatin-based adjuvant chemotherapy. Our patient remains free of disease to date.

**Conclusions:**

This case report sheds light on a rarely described disease and provides a comprehensive approach to diagnosis and management. Neuroendocrine carcinoma of the breast is a well-defined histologic subtype of breast cancer. Small cell carcinoma of the breast is a rare subtype of neuroendocrine breast cancer. Due to the rarity of this entity, prognosis has still not been well established, and treatment has not been standardized, cisplatin-based treatment has been used in this case similar to small cell carcinoma of the lung.

## Background

Neuroendocrine breast cancer (NEBC) is rare, with an incidence of 0.3 to 1% of all breast cancers [1, 2]. Although NEBC was first described more than 40 years ago, it was not until 2003 that the World Health Organization (WHO) defined NEBC as a separate subtype of breast cancer [3].

The diagnosis of NEBC should fulfill three criteria, according to the WHO definition of the disease. First, immunohistochemistry should identify neuroendocrine features in at least 50% of the tumor cells. Chromogranin and synaptophysin are the immunostains most used for NEBC diagnosis; CD56 and neuron-specific enolase (NSE) lack specificity for NEBC in mammary sites, especially because they are expressed in normal breast tissue [4]. In cases where neuroendocrine features are present in less than 50% of the tumor cells, the tumor should be classified as breast carcinoma with neuroendocrine differentiation. It should be noted that focal neuroendocrine differentiation within breast tumors is common and has no prognostic significance [5, 6]. The second criterion for the diagnosis of NEBC is the exclusion of extra-mammary primary tumors, mainly in the lungs and gastrointestinal tract, usually with the help of imaging techniques other than breast imaging such as a chest and abdomen computed tomography (CT) scanning or positron emission tomography (PET-CT) scanning. The third criterion is the presence of an *in situ* component on breast pathology. Certain architectural patterns such as papillary, nesting, or mixed should also raise suspicion about the possibility of NEBC [7].

The three histologic subtypes defined by the WHO are well-differentiated neuroendocrine tumors; poorly differentiated tumors, which include small cell carcinoma; and invasive carcinoma with neuroendocrine features.

Small cell carcinoma of the breast has been very rarely reported in the literature [8–11]. The largest population-based series, by Hare *et al*., included 199 patients with small cell carcinoma of the breast, who were compared with patients with small cell lung carcinoma [12].

We report a case of a premenopausal woman diagnosed with primary small cell carcinoma of the breast, summarizing the challenges associated with histogenesis, prognosis, and treatment in neuroendocrine tumors of the breast. This case report sheds light on a rarely described disease and provides a comprehensive approach to diagnosis and management.

## Case presentation

A 47-year-old white woman, from Beirut, Lebanon, presented for general surgery consultation for a suspicious left breast mass discovered on routine mammogram. Our patient was premenopausal and had given birth to two children whom she had breastfed for 6 months each. She had had no previous medical problems and no previous surgeries. She had no family history of malignancy. She said she used to take calcium and vitamin D supplementation and denied taking oral contraceptives.

Our patient had no medical complaints. On physical examination, there was a 3 cm mass in the medial middle aspect of her left breast, with no nipple retraction or discharge, no changes in the overlying skin, and no palpable axillary lymph nodes. A digital mammogram showed a 30×26×29 mm round mass at the junction of the inner quadrants of the left breast.

**Fig. 1:**
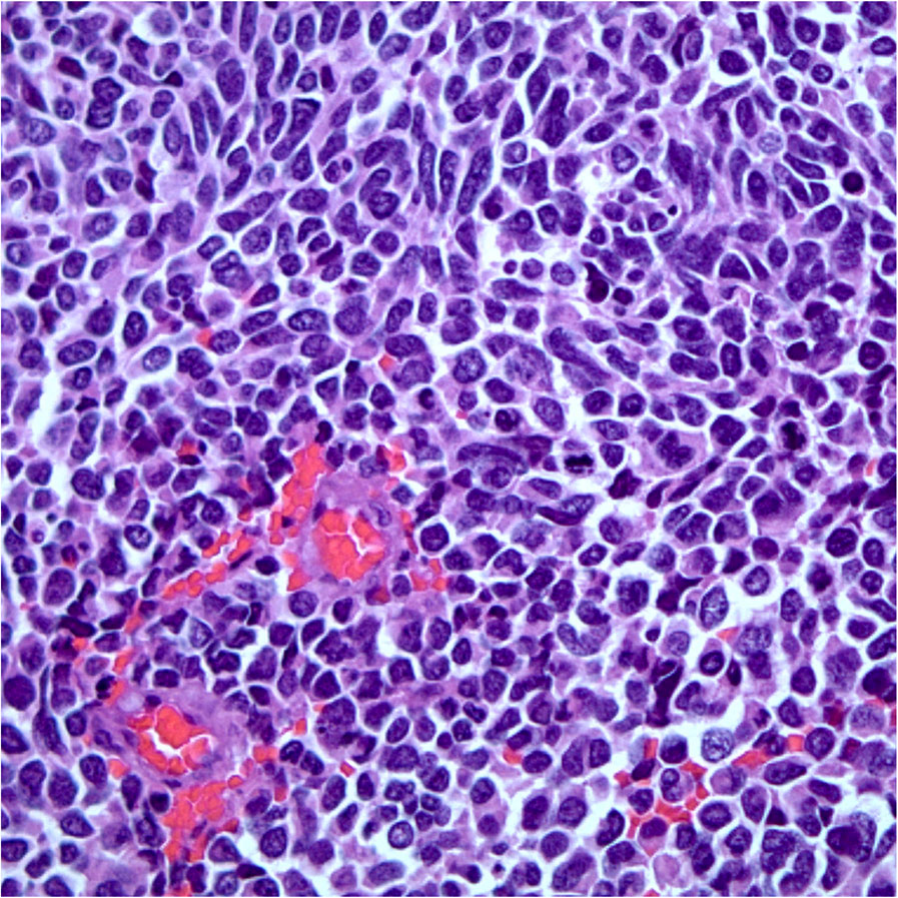
Core biopsy of the breast lesion. Image showing nuclear atypia with high nuclear to cytoplasmic ratio, nuclear molding, “salt and pepper” chromatin, and very brisk mitotic rate (hematoxylin and eosin, at high power field)

**Fig. 2:**
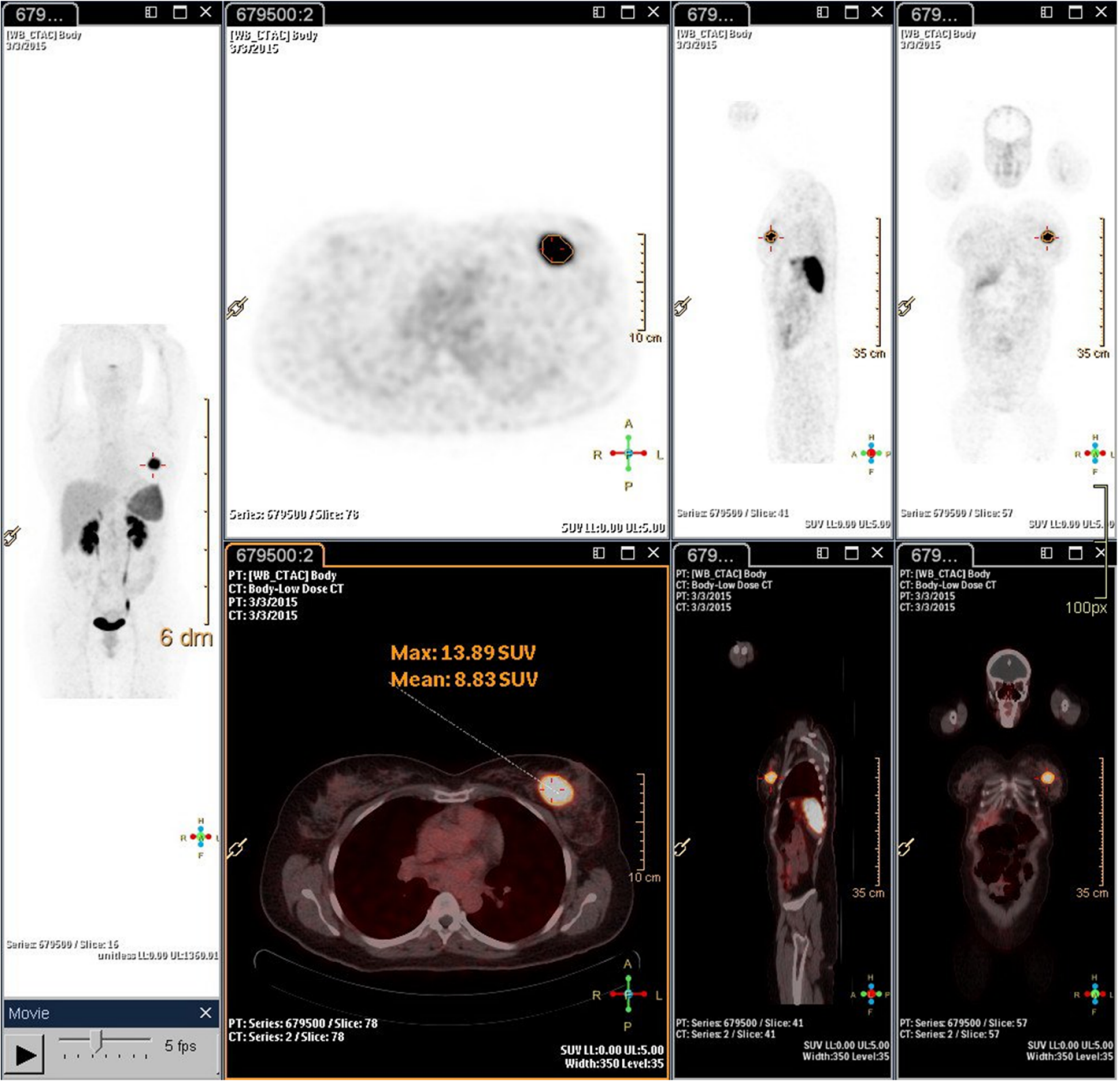
Positron emission tomography-computed tomography scan fused images for the whole body, showing the primary breast lesion and no extra-mammary involvement

An ultrasound-guided core biopsy of the lesion was performed, and it showed a small cell neuroendocrine tumor on pathology (Fig. 1). PET CT scan imaging and brain magnetic resonance imaging (MRI) were conducted to investigate the possibility of any other primary diseases, and the results were negative (Fig. 2).

**Fig. 3:**
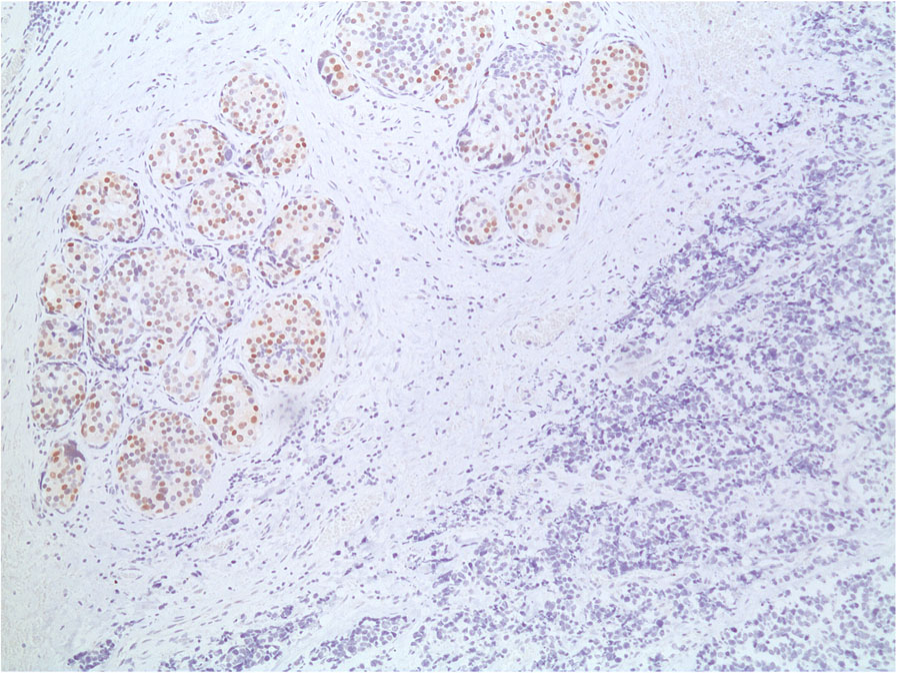
Image showing intermediate grade ductal carcinoma *in situ* lacking neuroendocrine features adjacent to the tumor, with differential staining between tumor and adjacent ductal carcinoma *in situ*. Estrogen receptor was not present in the tumor; ductal carcinoma *in situ* was estrogen receptor positive

**Fig. 4:**
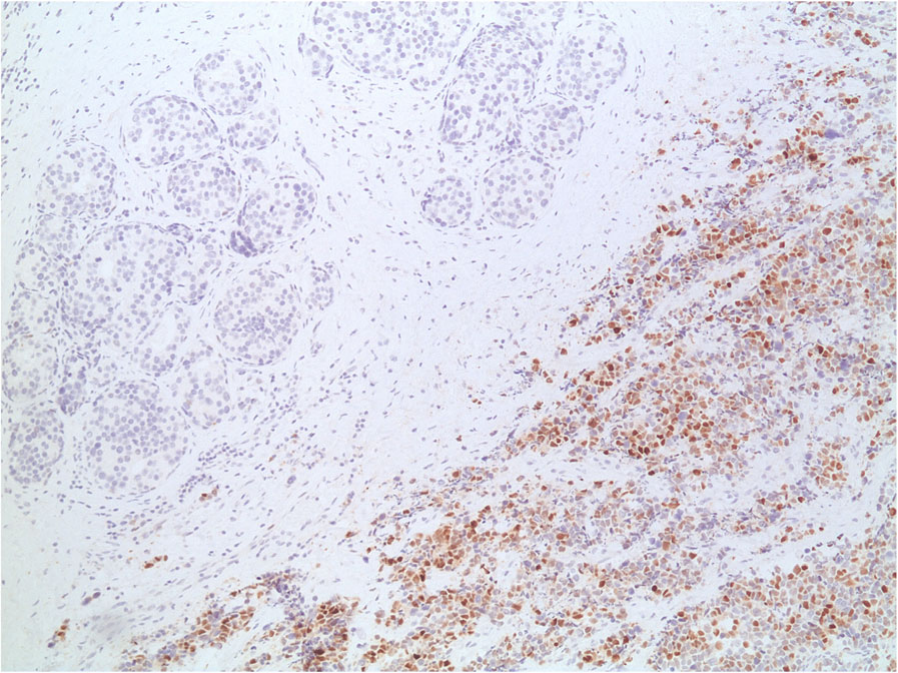
Differential staining between tumor and adjacent ductal carcinoma *in situ* with progesterone receptor present in 20% of the cells of the tumor and absent in ductal carcinoma *in situ*

**Fig. 5:**
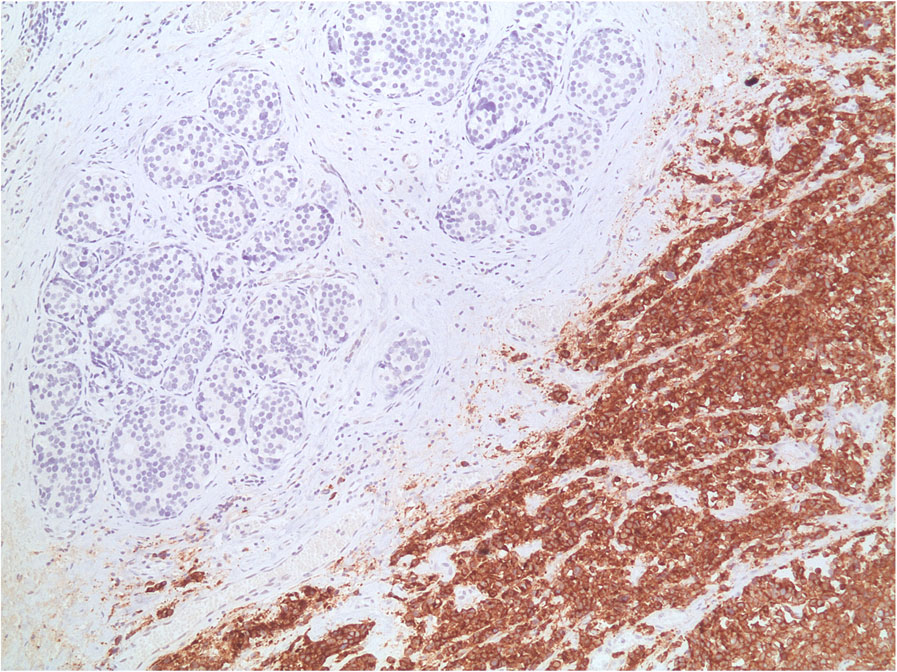
The breast lesion showing the tumor with synaptophysin staining that is positive in the tumor in more than 50% of the cells and absent in ductal carcinoma *in situ*

Our patient underwent a left total mastectomy and sentinel lymph node biopsy. Four sentinel nodes were retrieved using both the blue dye and the radioisotope methods. Pathology revealed a high-grade small cell neuroendocrine carcinoma, a Ki-67 proliferation index of 50%, estrogen receptor-negative disease (Fig. 3), 20% progesterone receptor-positive cells (Fig. 4), and negative Her2/neu. Chromogranin, CD56, and synaptophysin were all positive in more than 50% of tumor cells with evidence of ductal carcinoma *in situ* (Fig. 5). The surgical margins were free of tumor, and the four sentinel lymph nodes were negative. Our patient was diagnosed with small cell carcinoma of the breast (stage IIA), and she subsequently received adjuvant chemotherapy with four cycles of cisplatin 80 mg/m^2^ on day 1 and etoposide 100 mg/m^2^ for 3 days, followed by four cycles of 5-fluorouracil (5FU) 500 mg/m^2^, epirubicin 75 mg/m^2^, and cyclophosphamide 500 mg/m^2^ (FEC).

Our patient has received adjuvant hormonal treatment with tamoxifen for 10 months so far. She is on regular follow-up in the cancer center, and she remains free of disease to date.

## Discussion

Our patient was diagnosed with a primary small cell carcinoma of the breast, a rare entity where prognosis and treatment are still not well established. To help clinicians to recognize similar cases in their clinical practice and make a treatment plan, we discuss histogenesis, prognostic factors, and treatment options, including chemotherapy, radiation therapy, and hormonal therapy.

Small cell carcinoma of the breast resembles small cell carcinoma of the lung morphologically and immunohistochemically. Many hypotheses have tried to explain the histogenesis of neuroendocrine cells in mammary tissues; the most recent one is that NEBC is derived from divergent differentiation of a neoplastic stem cell into both epithelial and neuroendocrine cells [7]. Two older hypotheses suggested that NEBC is derived from neural crest cells that migrate to the mammary glands [13] or that it originates from neuroendocrine cells present in the breast tissue.

There have been many conflicting reports on the prognosis of NEBC: some have observed that NEBC may be less aggressive than the usual invasive ductal carcinoma subtype (IDC), [1, 14] and others have concluded that invasive neuroendocrine carcinoma of the breast has a poorer prognosis [15] than IDC. High nuclear grade, large tumor size, and regional lymph node metastasis have been identified as significant negative prognostic factors for distant recurrence-free survival [15]. Others have reported that the prognosis for NEBC is the same as that for other invasive breast cancers and is dependent on the staging, grading, mucin production, and apocrine differentiation of the tumor [5, 16, 17]. The small cell subtype of NEBC is associated with a very poor prognosis [18]; the proliferation rate (Ki-67 expression) is an independent prognostic factor of disease-free survival [19]. Next-generation sequencing of 19 cases of small cell carcinomas of the breast has revealed that the TP53 mutation is present in 75% of cases, correlating with the poor prognostic profile, and that the PIK3CA mutation is present in 33% of cases, representing a potential target for newly developed drugs [20].

Current therapy is increasingly based on gene expression profiling, and most cases of NEBC are hormone receptor positive and Her2/neu negative, which classifies them as belonging to the luminal subtype [21]. Interestingly, it has been shown that genes involved in migration, invasion, and proliferation are downregulated in this subtype, which gives NEBC a low potential for metastasis and means that it can be clustered with the mucinous carcinoma subtypes [21].

The management of such a rare histologic subtype of breast cancer poses a real challenge in daily clinical practice. Most of the information currently available to guide clinicians comes from case series or case reports, which means that it is very difficult to make a clear recommendation on how to manage neuroendocrine carcinoma of the breast. Surgical treatment with conserving breast surgeries, similar to the treatment of invasive ductal carcinoma, is the mainstay of therapy.

There is no uniform strategy on how to treat this subtype of breast cancer with chemotherapy. Regimens used in the ductal subtype can be used in the same way in NEBC [22]. Some studies have showed benefits in using anthracycline-based chemotherapy in NEBC [23], whereas others have not [2, 15]. Some reports have recommended the use of anthracycline-based chemotherapy according to Ki-67 expression, usually if Ki-67 is around 10%. For poorly differentiated carcinomas, especially the small cell subtype where Ki-67 is more than 15%, the use of cisplatin and etoposide is recommended, as in small cell pulmonary tumors [24, 25]. Our patient had a very high Ki-67, reaching 50%, and she was treated with both cisplatin- and anthracycline-based chemotherapy.

Hormonal treatment in the adjuvant setting is given according to hormonal status. Most cases are estrogen receptor or progesterone receptor positive, and none of the tumors reported to date have been Her2/neu positive [15, 26]. Some reports have used somatostatin analogs in the adjuvant setting [7]. Our patient is receiving hormonal treatment with tamoxifen since progesterone receptors were present in 20% of the tumor cells. The role of radiotherapy in the treatment of NEBC is also controversial; it can resemble the algorithm used in ductal carcinoma with some benefit in overall survival [15]. However, Hare *et al*. reported exclusively on small cell carcinoma and did not find any benefit from radiation therapy [12].

## Conclusions

NEBC is a well-defined histologic subtype of breast cancer. Small cell carcinoma of the breast is a rare subtype of NEBC. Due to the rarity of NEBC, prognosis has still not been well established, and treatment has not been standardized. It is not feasible to conduct randomized multicenter clinical trials to assess the efficacy of chemotherapeutic agents and related long-term outcomes because of the limited number of patients diagnosed with NEBC. Reports on prognosis and treatment have largely involved comparing the small cell subtype with small cell carcinoma of the lung.

## Abbreviations

5FU: 5-fluorouracil
CT: Computed tomography
IDC: Invasive ductal carcinoma
Ki-67: Proliferation index
MRI: Magnetic resonance imaging
NEBC: Neuroendocrine breast cancer
NSE: Neuron-specific enolase
PET: Positron emission tomography
WHO: World Health Organization
